# 2-(4-Amino­phen­yl)-3,4,5,6-tetra­hydro­pyrimidin-1-ium chloride

**DOI:** 10.1107/S1600536812014493

**Published:** 2012-04-13

**Authors:** Krešimir Molčanov, Ivana Stolić, Biserka Kojić-Prodić, Goran Kovačević, Miroslav Bajić

**Affiliations:** a Department of Phyiscal Chemistry, Rudjer Bošković Institute, POB-180, HR-10002 Zagreb, Croatia; b Department of Chemistry and Biochemistry, Faculty of Veterinary Medicine, University of Zagreb, Heinzelova 55, HR-10000 Zagreb, Croatia

## Abstract

In the title compound, C_10_H_14_N_3_^+^·Cl^−^, the tetra­hydro­pyridinium ring of the cation, which adopts a slightly distorted envelope conformation, is disordered over two orientations with an occupancy ratio of 0.653 (5):0.347 (5). The amidinium fragment of the major conformer is twisted relative to the benzene ring by 22.5 (6)° and the two C—N bond lengths of this fragment are similar [1.3228 (16) and 1.319 (2) Å]. In the crystal, the chloride anions are involved in three N—H⋯Cl hydrogen bonds, which link the components into a two-dimensional hydrogen-bonded network parallel to (010).

## Related literature

For the synthesis, see: Wydra *et al.* (1990 ▶); Stolić *et al.* (2009 ▶, 2011 ▶). For related compounds, see: Molčanov *et al.* (2011 ▶); Jarak *et al.* (2005 ▶); Legrand *et al.* (2008 ▶). For the biological activity of compounds comprising a cyclic amidine system, see: Boykin (2002 ▶); Chaires *et al.* (2004 ▶); Farahat *et al.* (2011 ▶); Hall *et al.* (1998 ▶). For the *GAMESS* program package, see: Schmidt *et al.* (1993 ▶).
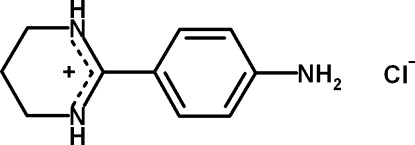


## Experimental

### 

#### Crystal data

C_10_H_14_N_3_^+^·Cl^−^
*M* *_r_* = 211.69
Orthorhombic, 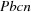

*a* = 15.0055 (2) Å
*b* = 8.0884 (1) Å
*c* = 17.8088 (3) Å
*V* = 2161.46 (5) Å^3^
*Z* = 8
Cu *K*α radiation
μ = 2.84 mm^−1^
*T* = 293 K
0.15 × 0.10 × 0.09 mm

#### Data collection

Oxford Diffraction Xcalibur Nova R diffractometer
Absorption correction: multi-scan (*CrysAlis PRO*; Agilent, 2011 ▶) *T*_min_ = 0.676, *T*_max_ = 0.784
6131 measured reflections
2242 independent reflections
1760 reflections with *I* > 2σ(*I*)
*R*_int_ = 0.017

#### Refinement

*R*[*F* ^2^ > 2σ(*F* ^2^)] = 0.031
*wR*(*F* ^2^) = 0.098
*S* = 1.05
2242 reflections
146 parameters
H-atom parameters constrained
Δρ_max_ = 0.18 e Å^−3^
Δρ_min_ = −0.16 e Å^−3^

### 

Data collection: *CrysAlis PRO* (Agilent, 2011 ▶); cell refinement: *CrysAlis PRO*; data reduction: *CrysAlis PRO*; program(s) used to solve structure: *SHELXS97* (Sheldrick, 2008 ▶); program(s) used to refine structure: *SHELXL97* (Sheldrick, 2008 ▶); molecular graphics: *ORTEP-3 for Windows* (Farrugia, 1997 ▶); software used to prepare material for publication: *WinGX* (Farrugia, 1999 ▶).

## Supplementary Material

Crystal structure: contains datablock(s) global, I. DOI: 10.1107/S1600536812014493/gk2441sup1.cif

Structure factors: contains datablock(s) I. DOI: 10.1107/S1600536812014493/gk2441Isup2.hkl

Supplementary material file. DOI: 10.1107/S1600536812014493/gk2441Isup3.cml

Additional supplementary materials:  crystallographic information; 3D view; checkCIF report

## Figures and Tables

**Table 1:** Hydrogen-bond geometry (Å, °)

*D*—H⋯*A*	*D*—H	H⋯*A*	*D*⋯*A*	*D*—H⋯*A*
N1—H1*NA*⋯Cl1^i^	0.90	2.47	3.3271 (16)	160
N2*B*—H2*N*⋯Cl1	0.90	2.27	3.1126 (12)	156
N3*B*—H3*N*⋯Cl1^ii^	0.90	2.42	3.250 (17)	153

Symmetry codes: (i) 
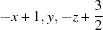
; (ii) 
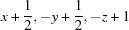
.

**Fig. 1.**
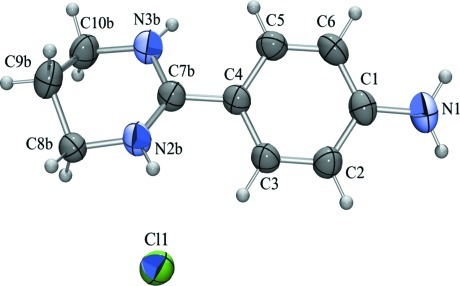
ORTEP-3 (Farrugia, 1997) drawing of the asymmetric unit of the title compound showing the major position of the disordered tetrahydropyrimidinium ring (B). Displacement ellipsoids are drawn at the 50% probability level and hydrogen atoms are depicted as spheres of arbitrary radii.

**Fig. 2.**
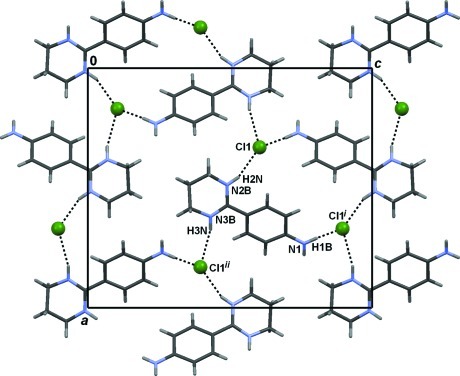
Hydrogen bonded (0 1 0) layer in the title compound. Symmetry operators: (i) x + 3/2, -y + 1/2, -z + 1; (ii) -x, y, -z + 3/2.

**Fig. 3.**
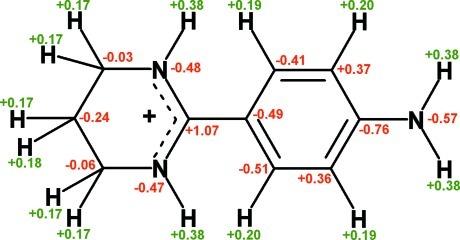
Mulliken charges calculated by DFT method.

## supplementary crystallographic information

### Comment

Natural and synthetic aromatic amidines that bind in the DNA minor groove have proved to be clinically useful agents primarly as antiparasitic agents.(Boykin, 2002; Farahat *et al.*, 2011). In addition to their antiparasitic properties, certain diamidines display a useful spectrum of antitumor, antiviral and antifungal activities. (Chaires *et al.*, 2004; Hall *et al.*, 1998; Stolić *et al.* 2009; Stolić *et al.* 2011). Cyclic amidine moiety is known in a number of potential antitumor agents; some of them have 4-(1,4,5,6-tetrahydropirimidin-2-yl)phenylamine as the building unit (Stolić *et al.*, 2011; Molčanov *et al.*, 2011).

The asymmetric unit contains a single formula unit of 2-(4-aminophenyl)-3,4,5,6-tetrahydropyrimidin-1-ium chloride. The tetrahydropyrimidinium ring is disordered over two positions designated as A and B (Fig. 1). Their respective occupancies are 0.347 (5)/ 0.653 (5). While there is a formal double C=N bond in the neutral tetrahydropyrimidine, both C—N bonds in the cation are approximately equal. However, the positive charge is localized on the C7 atom, as confirmed by DFT calculations (Fig. 3). Such a delocalization poses a significant restraint to conformation of the tetrahydropyrimidine ring. Cremer-Pople puckering parameters are Q = 0.454 (5)°, Θ = 132.8 (6)°, Φ = 55.2 (9)° for the conformer A (atom sequence N2B–C7B–N3B–C10B–C9B–C8B) and Q = 0.527 (12)°, Θ = 51.4 (11)°, Φ = 197.7 (13)° for the conformer B (atom sequence N2B–C7B–N3B–C10B–C9B–C8B) indicating the envelope form for the A ring and the conformation intermediate between half-chair and boat for the ring B. The chloride anions are coordinated by three N—H···Cl hydrogen bonds in the pyramidal arrangement. The crystal packing is dominated by hydrogen bonded layers parallel to (0 1 0) (Fig. 2, Table 1)

### Experimental

The crude imidate ester hydrochloride (1.61 g, 8.6 mmol) prepared from 4-aminobenzonitrile (1.12 g, 9.5 mmol) in anhydrous methanol by Pinner reaction was suspended in anhydrous methanol (100 ml), 1,3-diaminopropane (4 ml) was added and mixture was stirred at room temperature for 4 days under the nitrogen atmosphere. The solvent was removed under reduced pressure and residue was recrystallized from ethanol-diethyl ether to yield 1.06 g (57.6%) of white powder, IR (ν_max_/cm^-1^): 2883, 2023, 1595, 1472, 1101, 727, 635; ^1^H NMR (DMSO-d6) δ/p.p.m.: 8.28 (s, 2H, NH), 7.54 (d, 2H, J = 8.5 Hz, ArH), 6.65 (d, 2H, J = 8.1 Hz, ArH), 6.10 (s, 2H, NH_2_), 2.88 (t, 4H, J = 7.4 Hz, CH_2_), 1.90 (m, 2H, J = 7.4 Hz, CH_2_).

DFT calculations. The structure, obtained from the X-ray structural analysis was optimized without symmetry constrains by using MP2/6–31+G(d,p) level of theory implemented in the GAMESS program package (Schmidt *et al.*, 1993) . Tight convergence criteria were used in the optimization. The calculation was checked for convergence and frequencies were calculated in order to prove that the optimized structure was the minimum. The optimized geometry shows agreement with experimental one (conformer B); only four bonds (N1—C1, N3—C7, C2—C3 and C5–C6) differ more than 3 e.s.d.'s.

### Refinement

Hydrogen atoms were located from a difference Fourier map and refined as riding on their parent atoms. C—H bond lenghts were constrained to 0.93 and 0.97 Å for aromatic and methylene H atoms, respectively, while N—H bonds were constrained to 0.90 Å; *U*_iso_(H) = 1.2 *U*_eq_(C,*N*). Since the disordered atoms are very close to each other, they were refined with equal displacement ellipsoids using the command EADP in *SHELXL97* (Sheldrick, 2008) for every pair of disordered atoms (A and B), except C9A and C9B which had their displacement parameters refined independently.

### Crystal data

**Table:**

C_10_H_14_N_3_^+^·Cl^−^	*F*(000) = 896
*M**_r_* = 211.69	*D*_x_ = 1.301 Mg m^−^^3^
Orthorhombic, *P**b**c**n*	Cu *K*α radiation, λ = 1.54184 Å
Hall symbol: -P 2n 2ab	Cell parameters from 3458 reflections
*a* = 15.0055 (2) Å	θ = 2.5–76.0°
*b* = 8.0884 (1) Å	µ = 2.84 mm^−^^1^
*c* = 17.8088 (3) Å	*T* = 293 K
*V* = 2161.46 (5) Å^3^	Prism, colourless
*Z* = 8	0.15 × 0.10 × 0.09 mm

### Data collection

**Table:**

Oxford Diffraction Xcalibur Nova R diffractometer	1760 reflections with *I* > 2σ(*I*)
ω scans	*R*_int_ = 0.017
Absorption correction: multi-scan (*CrysAlis PRO*; Agilent, 2011)	θ_max_ = 76.2°, θ_min_ = 5.0°
*T*_min_ = 0.676, *T*_max_ = 0.784	*h* = −18→18
6131 measured reflections	*k* = −5→10
2242 independent reflections	*l* = −22→22

### Refinement

**Table:**

Refinement on *F*^2^	0 restraints
Least-squares matrix: full	H-atom parameters constrained
*R*[*F*^2^ > 2σ(*F*^2^)] = 0.031	*w* = 1/[σ^2^(*F*_o_^2^) + (0.0642*P*)^2^ + 0.0224*P*] where *P* = (*F*_o_^2^ + 2*F*_c_^2^)/3
*wR*(*F*^2^) = 0.098	(Δ/σ)_max_ = 0.001
*S* = 1.05	Δρ_max_ = 0.18 e Å^−^^3^
2242 reflections	Δρ_min_ = −0.16 e Å^−^^3^
146 parameters	

### Special details

**Table:**

Geometry. All e.s.d.'s (except the e.s.d. in the dihedral angle between two l.s. planes) are estimated using the full covariance matrix. The cell e.s.d.'s are taken into account individually in the estimation of e.s.d.'s in distances, angles and torsion angles; correlations between e.s.d.'s in cell parameters are only used when they are defined by crystal symmetry. An approximate (isotropic) treatment of cell e.s.d.'s is used for estimating e.s.d.'s involving l.s. planes.

### Fractional atomic coordinates and isotropic or equivalent isotropic displacement parameters (Å^2^)

**Table:**

	*x*	*y*	*z*	*U*_iso_*/*U*_eq_	Occ. (<1)
Cl1	0.33023 (2)	0.24010 (5)	0.60412 (2)	0.05806 (15)	
N1	0.73148 (10)	0.50143 (18)	0.76130 (9)	0.0710 (4)	
H1NB	0.7775	0.5727	0.7593	0.085*	
H1NA	0.7154	0.4539	0.8049	0.085*	
N2A	0.48706 (7)	0.24901 (14)	0.48980 (7)	0.0481 (3)	0.347 (5)
H2NB	0.4516	0.2692	0.5296	0.058*	0.347 (5)
N2B	0.48706 (7)	0.24901 (14)	0.48980 (7)	0.0481 (3)	0.653 (5)
H2N	0.4516	0.2691	0.5295	0.058*	0.653 (5)
N3A	0.6227 (6)	0.2596 (6)	0.4311 (5)	0.0498 (10)	0.653 (5)
H3M	0.682	0.2722	0.4359	0.06*	0.653 (5)
N3B	0.6181 (11)	0.3005 (15)	0.4279 (11)	0.0498 (10)	0.347 (5)
H3N	0.6774	0.3132	0.4327	0.06*	0.347 (5)
C1	0.69335 (9)	0.45272 (15)	0.69569 (8)	0.0474 (3)	
C2	0.61748 (9)	0.35056 (16)	0.69653 (7)	0.0475 (3)	
H2	0.5937	0.3163	0.7422	0.057*	
C3	0.57817 (8)	0.30093 (16)	0.63062 (7)	0.0436 (3)	
H3	0.5275	0.2348	0.6323	0.052*	
C4	0.61292 (8)	0.34788 (14)	0.56100 (7)	0.0405 (3)	
C5	0.68797 (9)	0.45061 (16)	0.56016 (8)	0.0483 (3)	
H5	0.712	0.4839	0.5145	0.058*	
C6	0.72660 (10)	0.50288 (16)	0.62576 (9)	0.0510 (3)	
H6	0.7757	0.5729	0.6238	0.061*	
C7A	0.57250 (8)	0.28921 (15)	0.49100 (7)	0.0427 (3)	0.347 (5)
C7B	0.57250 (8)	0.28921 (15)	0.49100 (7)	0.0427 (3)	0.653 (5)
C8A	0.4451 (10)	0.2003 (13)	0.4167 (7)	0.0457 (8)	0.347 (5)
H8A1	0.4026	0.2777	0.3957	0.055*	0.347 (5)
H8A2	0.4127	0.1021	0.4324	0.055*	0.347 (5)
C8B	0.4427 (5)	0.1637 (5)	0.4285 (3)	0.0457 (8)	0.653 (5)
H8A	0.446	0.0442	0.432	0.055*	0.653 (5)
H8B	0.3803	0.1951	0.4276	0.055*	0.653 (5)
C9A	0.5137 (3)	0.1287 (7)	0.3654 (2)	0.0539 (14)	0.347 (5)
H9A1	0.5428	0.0266	0.3802	0.065*	0.347 (5)
H9A2	0.4832	0.1087	0.3183	0.065*	0.347 (5)
C9B	0.48604 (17)	0.2297 (4)	0.35639 (14)	0.0574 (8)	0.653 (5)
H9B1	0.475	0.3468	0.3492	0.069*	0.653 (5)
H9B2	0.4611	0.1709	0.3138	0.069*	0.653 (5)
C10A	0.5833 (7)	0.2609 (10)	0.3539 (6)	0.0575 (9)	0.347 (5)
H10C	0.6269	0.2273	0.3167	0.069*	0.347 (5)
H10D	0.5567	0.3649	0.3386	0.069*	0.347 (5)
C10B	0.5866 (3)	0.1982 (5)	0.3599 (3)	0.0575 (9)	0.653 (5)
H10A	0.5985	0.0804	0.3593	0.069*	0.653 (5)
H10B	0.6159	0.2482	0.317	0.069*	0.653 (5)

### Atomic displacement parameters (Å^2^)

**Table:**

	*U*^11^	*U*^22^	*U*^33^	*U*^12^	*U*^13^	*U*^23^
Cl1	0.0367 (2)	0.0877 (3)	0.0498 (2)	−0.00153 (14)	0.00414 (13)	0.00525 (16)
N1	0.0747 (9)	0.0785 (8)	0.0598 (8)	−0.0183 (7)	−0.0182 (7)	−0.0077 (6)
N2A	0.0334 (5)	0.0656 (7)	0.0454 (6)	0.0000 (4)	0.0010 (5)	−0.0087 (5)
N2B	0.0334 (5)	0.0656 (7)	0.0454 (6)	0.0000 (4)	0.0010 (5)	−0.0087 (5)
N3A	0.0340 (10)	0.069 (3)	0.0461 (10)	0.005 (2)	0.0034 (8)	−0.006 (2)
N3B	0.0340 (10)	0.069 (3)	0.0461 (10)	0.005 (2)	0.0034 (8)	−0.006 (2)
C1	0.0431 (6)	0.0459 (6)	0.0533 (7)	0.0034 (5)	−0.0080 (6)	−0.0059 (5)
C2	0.0433 (7)	0.0561 (7)	0.0430 (6)	−0.0005 (5)	0.0012 (6)	−0.0006 (6)
C3	0.0350 (6)	0.0488 (6)	0.0470 (7)	−0.0029 (5)	0.0009 (5)	−0.0013 (5)
C4	0.0322 (5)	0.0453 (6)	0.0440 (6)	0.0024 (4)	0.0000 (5)	−0.0004 (5)
C5	0.0407 (6)	0.0529 (7)	0.0515 (7)	−0.0045 (5)	0.0037 (6)	0.0042 (5)
C6	0.0395 (6)	0.0508 (7)	0.0627 (8)	−0.0083 (5)	−0.0033 (6)	−0.0019 (6)
C7A	0.0354 (6)	0.0495 (6)	0.0433 (6)	0.0037 (5)	0.0008 (5)	−0.0003 (5)
C7B	0.0354 (6)	0.0495 (6)	0.0433 (6)	0.0037 (5)	0.0008 (5)	−0.0003 (5)
C8A	0.0416 (7)	0.050 (2)	0.0454 (19)	−0.0047 (19)	−0.0044 (13)	0.0034 (14)
C8B	0.0416 (7)	0.050 (2)	0.0454 (19)	−0.0047 (19)	−0.0044 (13)	0.0034 (14)
C9A	0.052 (3)	0.061 (3)	0.049 (2)	0.005 (2)	−0.0064 (19)	−0.0088 (19)
C9B	0.0558 (14)	0.0712 (18)	0.0453 (12)	0.0074 (12)	−0.0077 (10)	−0.0036 (11)
C10A	0.0534 (10)	0.077 (3)	0.0425 (11)	0.004 (2)	0.0041 (9)	−0.007 (2)
C10B	0.0534 (10)	0.077 (3)	0.0425 (11)	0.004 (2)	0.0041 (9)	−0.007 (2)

### Geometric parameters (Å, º)

**Table:**

N1—C1	1.3593 (18)	C4—C5	1.3996 (17)
N1—H1NB	0.8999	C4—C7A	1.4653 (17)
N1—H1NA	0.8999	C5—C6	1.3710 (19)
N2A—C7A	1.3228 (17)	C5—H5	0.93
N2A—C8A	1.499 (14)	C6—H6	0.93
N2A—H2NB	0.901	C8A—C9A	1.494 (15)
N2B—H2N	0.9	C8A—H8A1	0.9677
N2B—C7B	1.3228 (16)	C8A—H8A2	0.9717
N2B—C8B	1.453 (6)	C8B—C9B	1.535 (6)
N3A—C7A	1.327 (9)	C8B—H8A	0.97
N3A—C10A	1.497 (14)	C8B—H8B	0.97
N3A—H3M	0.8999	C9A—C10A	1.509 (11)
N3B—C10B	1.541 (19)	C9A—H9A1	0.97
N3B—C7B	1.319 (2)	C9A—H9A2	0.97
N3B—H3N	0.8999	C9B—C10B	1.531 (6)
C1—C6	1.402 (2)	C9B—H9B1	0.97
C1—C2	1.4069 (19)	C10A—H10C	0.97
C2—C3	1.3736 (18)	C10A—H10D	0.97
C2—H2	0.93	C10B—H10A	0.97
C3—C4	1.3975 (18)	C10B—H10B	0.97
C3—H3	0.93		
			
C1—N1—H1NB	118.4	C10A—C9A—H9A2	109.1
C1—N1—H1NA	120.4	H9A1—C9A—H9A2	107.8
H1NB—N1—H1NA	120.9	C8A—C9A—H9B2	84.7
C7A—N2A—C8A	119.1 (6)	C10A—C9A—H9B2	98.2
C7A—N2A—H2NB	121	H9A1—C9A—H9B2	135.3
C8A—N2A—H2NB	118.8	C8A—C9A—H10A	145.1
C7A—N2A—H2N	121	C10A—C9A—H10A	62.4
C8A—N2A—H2N	118.8	H9A1—C9A—H10A	49
C7A—N3A—C10A	120.9 (7)	H9A2—C9A—H10A	109.4
C7A—N3A—H3M	117.6	H9B2—C9A—H10A	128.2
C10A—N3A—H3M	118.5	C10B—C9B—C8B	109.0 (4)
C7A—N3A—H3N	113.1	C10B—C9B—H8A1	148.6
C10A—N3A—H3N	111.9	C8B—C9B—H8A1	48.7
C10B—N3B—H3M	106.6	C10B—C9B—H9A2	85.5
C10B—N3B—H3N	116.1	C8B—C9B—H9A2	100.1
N1—C1—C6	122.01 (13)	H8A1—C9B—H9A2	116.9
N1—C1—C2	120.11 (13)	C10B—C9B—H9B1	109.6
C6—C1—C2	117.87 (12)	C8B—C9B—H9B1	112.2
C3—C2—C1	120.67 (12)	H8A1—C9B—H9B1	70.4
C3—C2—H2	119.7	H9A2—C9B—H9B1	136.1
C1—C2—H2	119.7	C10B—C9B—H9B2	109.3
C2—C3—C4	121.22 (12)	C8B—C9B—H9B2	108.6
C2—C3—H3	119.4	H8A1—C9B—H9B2	99.8
C4—C3—H3	119.4	H9B1—C9B—H9B2	108.1
C3—C4—C5	118.10 (11)	C10B—C9B—H10D	56.9
C3—C4—C7A	120.81 (11)	C8B—C9B—H10D	134.7
C5—C4—C7A	121.08 (11)	H8A1—C9B—H10D	119.1
C6—C5—C4	120.94 (12)	H9A2—C9B—H10D	119.1
C6—C5—H5	119.5	H9B1—C9B—H10D	53.3
C4—C5—H5	119.5	H9B2—C9B—H10D	116.7
C5—C6—C1	121.16 (12)	N3A—C10A—C9A	98.2 (6)
C5—C6—H6	119.4	N3A—C10A—H10C	111.1
C1—C6—H6	119.4	C9A—C10A—H10C	111.2
N2A—C7A—N3A	119.5 (4)	N3A—C10A—H10D	115.2
N2A—C7A—C4	119.64 (12)	C9A—C10A—H10D	111.6
N3A—C7A—C4	120.5 (4)	H10C—C10A—H10D	109.3
C9A—C8A—N2A	110.1 (10)	N3A—C10A—H10A	82.6
C9A—C8A—H8A1	117.9	C9A—C10A—H10A	53
N2A—C8A—H8A1	116.2	H10C—C10A—H10A	70.4
C9A—C8A—H8A2	101.8	H10D—C10A—H10A	159.5
N2A—C8A—H8A2	100	N3A—C10A—H10B	119.8
H8A1—C8A—H8A2	108.1	C9A—C10A—H10B	115.4
C9A—C8A—H8A	75.1	H10D—C10A—H10B	97.5
N2A—C8A—H8A	94	H10A—C10A—H10B	80.6
H8A1—C8A—H8A	136.1	C9B—C10B—N3B	104.2 (7)
C9A—C8A—H8B	141.2	C9B—C10B—H9A1	75.3
N2A—C8A—H8B	104.6	N3B—C10B—H9A1	114.9
H8A1—C8A—H8B	57.2	C9B—C10B—H10C	121.5
H8A2—C8A—H8B	54.5	N3B—C10B—H10C	106.9
H8A—C8A—H8B	85.9	H9A1—C10B—H10C	129.1
C9B—C8B—H8A1	63.6	C9B—C10B—H10D	62.4
C9B—C8B—H8A2	128.9	N3B—C10B—H10D	78.7
H8A1—C8B—H8A2	105.9	H9A1—C10B—H10D	137.7
C9B—C8B—H8A	112.3	H10C—C10B—H10D	76.8
H8A1—C8B—H8A	142.5	C9B—C10B—H10A	110.2
C9B—C8B—H8B	107.8	N3B—C10B—H10A	118.6
H8A1—C8B—H8B	48.2	H10C—C10B—H10A	96.3
H8A2—C8B—H8B	63.4	H10D—C10B—H10A	162.7
H8A—C8B—H8B	108.2	C9B—C10B—H10B	110.2
C8A—C9A—C10A	106.6 (7)	N3B—C10B—H10B	104.8
C8A—C9A—H9A1	118.3	H9A1—C10B—H10B	137.3
C10A—C9A—H9A1	109.2	H10D—C10B—H10B	63.5
C8A—C9A—H9A2	105.5	H10A—C10B—H10B	108.4
			
N1—C1—C2—C3	179.79 (13)	C10A—N3A—C7A—N2A	29.8 (6)
C6—C1—C2—C3	0.59 (19)	C10A—N3A—C7A—C4	−157.3 (5)
C1—C2—C3—C4	1.0 (2)	C3—C4—C7A—N2A	26.62 (18)
C2—C3—C4—C5	−1.51 (19)	C5—C4—C7A—N2A	−154.22 (12)
C2—C3—C4—C7A	177.68 (12)	C3—C4—C7A—N3A	−146.3 (3)
C3—C4—C5—C6	0.38 (19)	C5—C4—C7A—N3A	32.9 (3)
C7A—C4—C5—C6	−178.81 (12)	C7A—N2A—C8A—C9A	27.2 (7)
C4—C5—C6—C1	1.3 (2)	N2A—C8A—C9A—C10A	−59.5 (8)
N1—C1—C6—C5	179.09 (13)	C7A—N3A—C10A—C9A	−58.3 (7)
C2—C1—C6—C5	−1.7 (2)	C8A—C9A—C10A—N3A	69.8 (8)
C8A—N2A—C7A—N3A	−11.2 (5)	C8B—C9B—C10B—N3B	−62.7 (7)
C8A—N2A—C7A—C4	175.9 (4)		

### Hydrogen-bond geometry (Å, º)

**Table:**

*D*—H···*A*	*D*—H	H···*A*	*D*···*A*	*D*—H···*A*
N1—H1*NA*···Cl1^i^	0.90	2.47	3.3271 (16)	160
N2*B*—H2*N*···Cl1	0.90	2.27	3.1126 (12)	156
N3*B*—H3*N*···Cl1^ii^	0.90	2.42	3.250 (17)	153

Symmetry codes: (i) −*x*+1, *y*, −*z*+3/2; (ii) *x*+1/2, −*y*+1/2, −*z*+1.

### Calculated bond lengths (Å)

**Table:**

N1	C1	1.372
N1	H1NB	1.008
N1	H1NA	1.008
N2	C7	1.332
N2	H2A	1.011
N2	C8	1.470
N3	C7	1.332
N3	C10	1.469
N3	H3	0.899
C1	C6	1.411
C1	C2	1.411
C2	C3	1.388
C2	H2	1.083
C3	C4	1.407
C3	H3	1.084
C4	C5	1.407
C4	C7	1.458
C5	C6	1.388
C5	H5	1.084
C6	H6	1.083
C8	C9	1.520
C8	H8A	1.089
C8	H8B	1.092
C9	C10	1.522
C9	H9A	1.089
C9	H9B	1.091
C10	H10A	1.087
C10	H10B	1.092
